# Treatment delay significantly increases mortality in colorectal cancer: a meta-analysis

**DOI:** 10.1007/s11357-025-01648-z

**Published:** 2025-04-08

**Authors:** Zoltan Ungvari, Mónika Fekete, János Tibor Fekete, Andrea Lehoczki, Annamaria Buda, Gyöngyi Munkácsy, Péter Varga, Anna Ungvari, Balázs Győrffy

**Affiliations:** 1 Vascular Cognitive Impairment, Neurodegeneration and Healthy Brain Aging Program, Department of Neurosurgery, University of Oklahoma Health Sciences Center, Oklahoma City, OK USA; 2 Stephenson Cancer Center, University of Oklahoma, Oklahoma City, OK USA; 3 Oklahoma Center for Geroscience and Healthy Brain Aging, University of Oklahoma Health Sciences Center, Oklahoma City, OK USA; 4 Department of Health Promotion Sciences, College of Public Health, University of Oklahoma Health Sciences Center, Oklahoma City, OK USA; 5 International Training Program in Geroscience, Doctoral College, Health Sciences Division/Institute of Preventive Medicine and Public Health, Semmelweis University, Budapest, Hungary; 6 Institute of Preventive Medicine and Public Health, Semmelweis University, Semmelweis University, Budapest, Hungary; 7 Jozsef Fodor Center for Prevention and Healthy Aging, Semmelweis University, Budapest, Hungary; 8 Dept. Of Bioinformatics, Semmelweis University, 1094 Budapest, Hungary; 9 Cancer Biomarker Research Group, Institute of Molecular Life Sciences, HUN-REN Research Centre for Natural Sciences, 1117 Budapest, Hungary; 10 Doctoral College, Health Sciences Division, Semmelweis University, Budapest, Hungary; 11 Dept. Of Biophysics, Medical School, University of Pecs, 7624 Pecs, Hungary

**Keywords:** Colorectal cancer, Treatment delay, Cancer survival, Oncology care pathways, Healthcare inefficiencies, Geriatric oncology, Frailty, Multimorbidity, Patient navigation, Referral pathways, Telemedicine, Diagnostic delays, Healthcare disparities, Time-to-treatment benchmarks, Cancer treatment access

## Abstract

Delaying the initiation of cancer treatment increases the risk of mortality, particularly in colorectal cancer (CRC), which is among the most common and deadliest malignancies. This study aims to explore the impact of treatment delays on mortality in CRC. A systematic literature search was conducted in PubMed, Web of Science, and Scopus for studies published between 2000 and 2025. Meta-analyses were performed using random-effects models with inverse variance method to calculate hazard ratios (HRs) for both overall and cancer-specific survival at 4-, 8-, and 12-week treatment delay intervals, with heterogeneity assessed through *I*^2^-statistics and publication bias evaluated using funnel plots and Egger’s test. A total of 20 relevant studies were included in the meta-analysis. The analyses of all patients demonstrated a progressively increasing risk of 12–39% with longer treatment delays (4 weeks, HR = 1.12; 95% CI, 1.08–1.16; 8 weeks, HR = 1.24; 95% CI, 1.16–1.34; 12 weeks, HR = 1.39; 95% CI, 1.25–1.55). In particular, incrementally higher hazard ratios were observed for all–cause mortality at 4 weeks (HR = 1.14; 95% CI, 1.09–1.18), 8 weeks (HR = 1.29; 95% CI, 1.20–1.39), and 12 weeks (HR = 1.47; 95% CI, 1.31–1.64). In contrast, cancer-specific survival analysis showed a similar trend but did not reach statistical significance (4 weeks, HR = 1.07; 95% CI, 0.98–1.18; 8 weeks, HR = 1.15; 95% CI, 0.95–1.39; 12 weeks, HR = 1.23; 95% CI, 0.93–1.63). Treatment delays in colorectal cancer patients were associated with progressively worsening overall survival, with each 4-week delay increment leading to a substantially higher mortality risk. This study suggests that timely treatment initiation should be prioritized in clinical practice, as these efforts can lead to substantial improvements in survival rates.

## Introduction

Timely initiation of cancer treatment is essential for optimizing survival outcomes, yet treatment delays remain a persistent challenge in oncology [1–4]. Colorectal cancer (CRC) is a disease of aging [5, 6], with the majority of cases occurring in individuals over 60 [7, 8]. Despite advances in screening and treatment, CRC remains one of the leading causes of cancer-related mortality worldwide [7], and growing evidence suggests that even modest delays in treatment initiation significantly worsen survival outcomes [4, 9]. However, delays in CRC treatment continue to occur due to a complex interplay of patient-, provider-, and healthcare system-related factors, underscoring the urgent need to better understand and mitigate these barriers [10].

Delays in CRC treatment arise at multiple levels [11–15]. Patient-related factors include low symptom awareness, misattribution of early symptoms to benign conditions, reluctance to seek medical attention, and logistical or financial constraints [12, 16–18]. Older patients, in particular, often face additional challenges such as frailty, cognitive impairment, and difficulties in accessing care. Healthcare system inefficiencies, including prolonged wait times for specialist referrals, limited access to diagnostic procedures (e.g., colonoscopies and imaging), and overburdened oncology services, further contribute to treatment delays [19–22]. For instance, in Great Britain, the proportion of cancer patients waiting over 104 days for treatment has increased nearly fourfold, from 6000 in 2016 to 22,000 in 2024 [23]. In 2024, only 62% of National Health Service patients started treatment within 62 days of an urgent suspected cancer referral [23]. Provider-related factors, such as misdiagnosis, delayed decision-making, and concerns over treatment tolerance—particularly in older or medically complex patients—can also extend time to treatment [24, 25]. Additionally, global crises such as the COVID- 19 pandemic have significantly disrupted cancer care delivery, leading to widespread postponements in surgery, chemotherapy, and radiotherapy [26–29].

The consequences of treatment delays in CRC are profound [4, 9], with studies suggesting that each additional week of delay increases the risk of mortality, likely due to continued tumor progression and reduced effectiveness of treatment interventions. While delays in surgery, chemotherapy, and radiation therapy have all been linked to poorer survival outcomes, there remains a lack of standardized benchmarks for defining acceptable treatment timelines. Addressing these delays is particularly crucial in aging populations, where cancer progression may be influenced by age-related declines in immune surveillance, systemic inflammation, and multimorbidity.

This study aims to provide a comprehensive meta-analysis quantifying the impact of treatment delays on both overall and cancer-specific mortality in CRC. By systematically analyzing data across multiple studies, we assess how incremental delays of 4, 8, and 12 weeks affect survival outcomes. Our findings underscore the critical need for timely treatment initiation, inform clinical decision-making, and highlight areas where healthcare policies can be optimized to improve cancer care delivery.

## Methods

### Data collection

In this meta-analysis, we aimed to investigate the relationship between delays in initiating treatment and mortality in colorectal cancer. We included different therapeutic approaches, including surgical intervention, systemic chemotherapy, and radiation therapy. To gather relevant literature, we conducted a systematic search across three major electronic databases: PubMed, Scopus, and Web of Science, covering publications from 2000 to 2025. Our search strategy combined relevant MeSH terms and keywords for colorectal cancer, treatment delays, and survival outcomes, ensuring comprehensive coverage of available studies. The definition of treatment delay varied across studies, but was most commonly reported as the time from diagnosis to surgery. A minority of studies assessed delays to systemic therapy or radiotherapy.

We applied strict inclusion and exclusion criteria to select studies for analysis. We included prospective and retrospective cohort studies that assessed the impact of treatment delays on mortality in colorectal cancer patients. Only studies reporting hazard ratios, odds ratios, or risk ratios to quantify this relationship and those with a minimum follow-up duration of 4 weeks were considered. Conversely, we excluded experimental studies involving animal models, in vitro investigations, or theoretical simulations. Non-English publications and studies with inadequate methodological rigor or improperly defined patient populations were also omitted. The study was conducted in accordance with key elements of the PRISMA 2020 guidelines. However, we did not submit a completed checklist, which we note as a limitation.

### Data extraction

Data extraction was carried out independently by two researchers, who systematically collected information on study characteristics such as author names, publication year, and study outcome. Additionally, we recorded details on outcome measures, and hazard ratios describing the association between treatment delays and survival outcomes. The duration and classification of treatment delays were also noted. In cases where discrepancies arose during data extraction, we resolved them through discussion until consensus was achieved. When agreement could not be reached, a third independent reviewer was consulted to ensure objectivity.

### Statistical analyses

We applied two different methods to calculate the hazard rate as an outcome variable. In the absence of a reference period, the reported HR/OR/RR values were standardized using the formula HR per *X*-month delay = (HR per 4 weeks delay)^(*X* weeks delay/4 weeks delay) [4]. For studies with a reported reference time, a weighted linear regression was applied to assess the association between treatment delay (weeks) and the log-transformed hazard ratio (HR) for patient outcomes. HR estimates with 95% confidence intervals were computed for delays of 4, 8, and 12 weeks.

To estimate aggregated risk measures, particularly the hazard ratios (HRs) with their corresponding 95% confidence intervals (CIs), we applied a random-effects model. This approach accounts for variations across studies, thereby enhancing the generalizability of the findings. A random-effects model was selected because the included studies differed in terms of population characteristics, definitions of delay, and healthcare settings—introducing clinical and methodological heterogeneity that warranted this modeling approach. To visualize individual study results alongside the overall summary estimate, we generated forest plots, which facilitated data interpretation and helped identify potential heterogeneity between studies. The statistical evaluation was conducted using the online platform MetaAnalysisOnline.com [30].

### Evaluation of variability and publication bias

To assess inter-study variability, we utilized Cochran’s *Q* test and the *I*^*2*^ statistic. Cochran’s *Q* test, based on a chi-squared distribution, was employed to determine whether observed differences in effect sizes exceeded those expected by random variation. Additionally, the *I*^*2*^ statistic quantified the proportion of total variance attributed to actual study differences rather than chance fluctuations.

To examine potential publication bias, we constructed funnel plots to graphically depict the relationship between study effect sizes and their precision. Any asymmetry in these plots could indicate the presence of bias. Furthermore, we performed Egger’s regression analysis to statistically evaluate the correlation between effect sizes and their standard errors, providing a quantitative measure of publication bias.

### Subgroup analyses

We conducted subgroup analyses to explore potential variations in effect estimates across different endpoints, including overall and cancer-specific survival. For each subgroup, we calculated pooled effect estimates along with heterogeneity metrics to evaluate the specific impact within each category. Additionally, we extended our analyses to the combined cohort, allowing for a comprehensive assessment of overall effects across all included conditions.

## Results

**Fig. 1:**
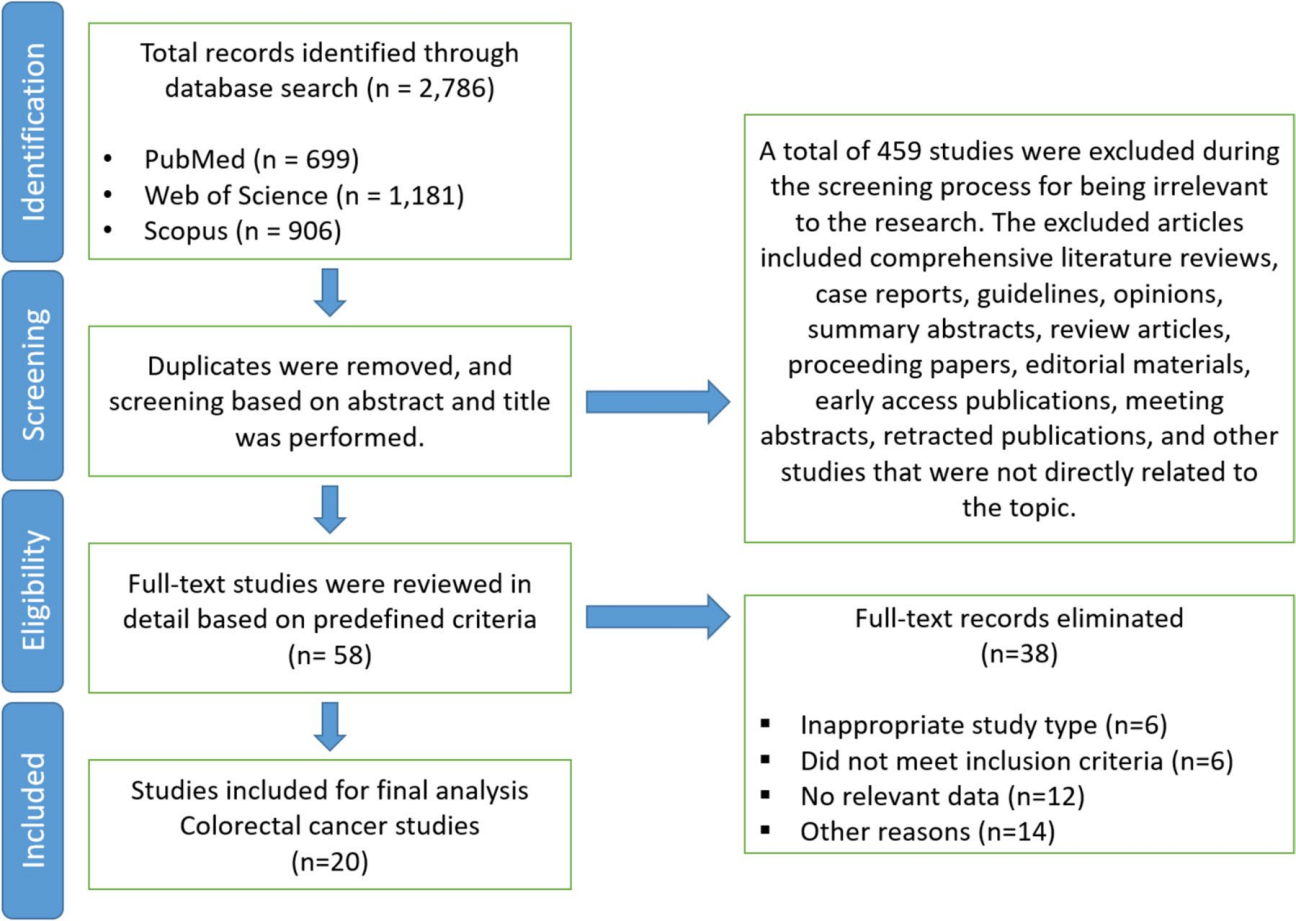
Flow diagram of study selection process

A total of 20 relevant studies were included in this meta-analysis [31–50]. Figure 1 presents the flowchart of the study selection process. The impact of treatment delays of 4, 8, and 12 weeks on survival outcomes in colorectal cancer patients were examined using the same set of studies—22 cohorts for overall survival, nine cohorts for colorectal cancer-specific mortality, and a total of 31 cohorts for the pooled analysis. Studies assessing the impact of delayed treatment on survival outcomes are summarized in Table 1.

**Table 1:** Summary of studies assessing the impact of delayed treatment on survival outcomes in colorectal cancer patients

Author	Year	Mortality	4 weeks delay			8 weeks delay			12 weeks delay			HR/OR/RR	Total *N*	Case *N*
			Rate	95% CI		Rate	95% CI		Rate	95% CI				
Ahmed et al	2010	All-cause	1.01	0.89	1.14	1.02	0.80	1.30	1.03	0.72	1.48	HR	663	275
Ahmed et al	2010	Cancer-specific	0.97	0.87	1.09	0.94	0.75	1.19	0.91	0.65	1.30	HR	663	275
Bagaria et al	2019	All-cause	1.08	1.06	1.10	1.17	1.13	1.22	1.27	1.20	1.34	OR	4685	2497
Bayraktar et al	2010	All-cause	1.40	1.01	1.95	1.97	1.02	3.81	2.77	1.03	7.43	HR	186	41
Bayraktar et al	2010	Cancer-specific	1.09	0.82	1.44	1.19	0.68	2.09	1.31	0.56	3.02	HR	186	32
Becerra et al	2017	All-cause	1.16	1.10	1.23	1.34	1.22	1.52	1.55	1.35	1.87	HR	1133	730
Becerra et al	2017	Cancer-specific	1.11	1.03	1.20	1.24	1.07	1.45	1.38	1.11	1.75	HR	1133	403
Chau et al	2005	All-cause	1.13	1.01	1.27	1.29	1.02	1.62	1.46	1.04	2.07	HR	801	220
Cheung et al	2009	All-cause	1.21	1.13	1.30	1.46	1.27	1.69	1.77	1.42	2.21	HR	6059	693
Cheung et al	2009	Cancer-specific	1.21	0.99	1.48	1.46	0.97	2.18	1.76	0.96	3.22	OR	6059	245
Edwards et al	2022	All-cause	1.01	0.81	1.27	1.03	0.65	1.60	1.04	0.52	2.03	HR	1031	167
Flemming et al	2017	All-cause	1.03	0.95	1.11	1.07	0.91	1.24	1.11	0.87	1.38	RR	4326	292
Flemming et al	2017	Cancer-specific	0.91	0.81	1.01	0.82	0.66	1.03	0.74	0.54	1.05	RR	4326	352
Hershman et al	2006	All-cause	1.15	1.10	1.20	1.32	1.21	1.44	1.52	1.33	1.73	HR	4382	665
Hershman et al	2006	Cancer-specific	1.03	0.88	1.19	1.05	0.78	1.41	1.08	0.69	1.68	HR	4382	665
Lima et al	2011	All-cause	1.21	1.11	1.30	1.45	1.24	1.70	1.75	1.38	2.22	HR	1053	475
Lima et al	2011	Cancer-specific	1.14	1.02	1.27	1.30	1.05	1.61	1.48	1.08	2.04	HR	1053	475
Lo et al	2021	All-cause	1.16	1.14	1.18	1.34	1.29	1.39	1.55	1.46	1.65	HR	187,394	6746
Massarweh et al	2015	All-cause	1.16	0.98	1.38	1.35	0.96	1.90	1.57	0.94	2.61	HR	51,331	16,570
Murchie et al	2014	All-cause	1.00	1.00	1.00	1.00	1.00	1.00	0.99	0.99	0.99	OR	958	762
Roder et al. (surgery)	2019	All-cause	1.05	0.98	1.13	1.11	0.96	1.28	1.16	0.94	1.45	HR	1675	1278
Roder et al. (radiotherapy)	2019	All-cause	1.14	1.06	1.22	1.29	1.11	1.49	1.46	1.18	1.82	HR	616	438
Roder et al. (systemic therapy)	2019	All-cause	1.01	0.99	1.02	1.01	0.98	1.04	1.02	0.97	1.07	HR	1556	1101
Roder et al. (any treatment)	2019	All-cause	1.11	0.95	1.29	1.23	0.91	1.66	1.36	0.86	2.14	HR	1675	1278
Shin et al	2013	All-cause	1.22	1.00	1.49	1.49	1.00	2.23	1.83	1.00	3.33	HR	7529	701
Strous et al	2019	All-cause	1.16	0.90	1.49	1.34	0.82	2.21	1.55	0.74	3.28	HR	790	179
Trepanier et al	2019	All-cause	1.62	1.08	2.42	2.61	1.16	5.88	4.22	1.25	14.26	HR	408	39
Trepanier et al	2019	Cancer-specific	0.93	0.90	0.96	0.86	0.81	0.92	0.80	0.73	0.88	HR	408	39
Xu et al	2014	All-cause	1.21	1.09	1.33	1.45	1.19	1.78	1.75	1.29	2.37	OR	4209	636
Xu et al	2014	Cancer-specific	1.62	1.30	2.02	2.62	1.69	4.07	4.23	2.19	8.20	OR	4209	464
Yun et al	2012	All-cause	1.42	1.27	1.59	2.03	1.61	2.52	2.89	2.04	4.01	HR	147,682	20,084
Zeig-Owens et al	2009	All-cause	1.10	0.95	1.27	1.20	0.90	1.61	1.32	0.86	2.05	HR	3006	1172

The table presents data extracted from individual studies, including author, year of publication, and subgroup classification (all-cause mortality or colorectal cancer-specific mortality). Reported hazard ratios, odds ratios, or risk ratios (HR/OR/RR) are provided for different treatment delay periods (4, 8, and 12 weeks), along with corresponding confidence intervals (lower and upper bounds)

*CI* confidence interval, *HR* hazard ratio, *OR* odds ratio, *RR* risk ratio

### Effect of 4-week delay in treatment

**Fig. 2:**
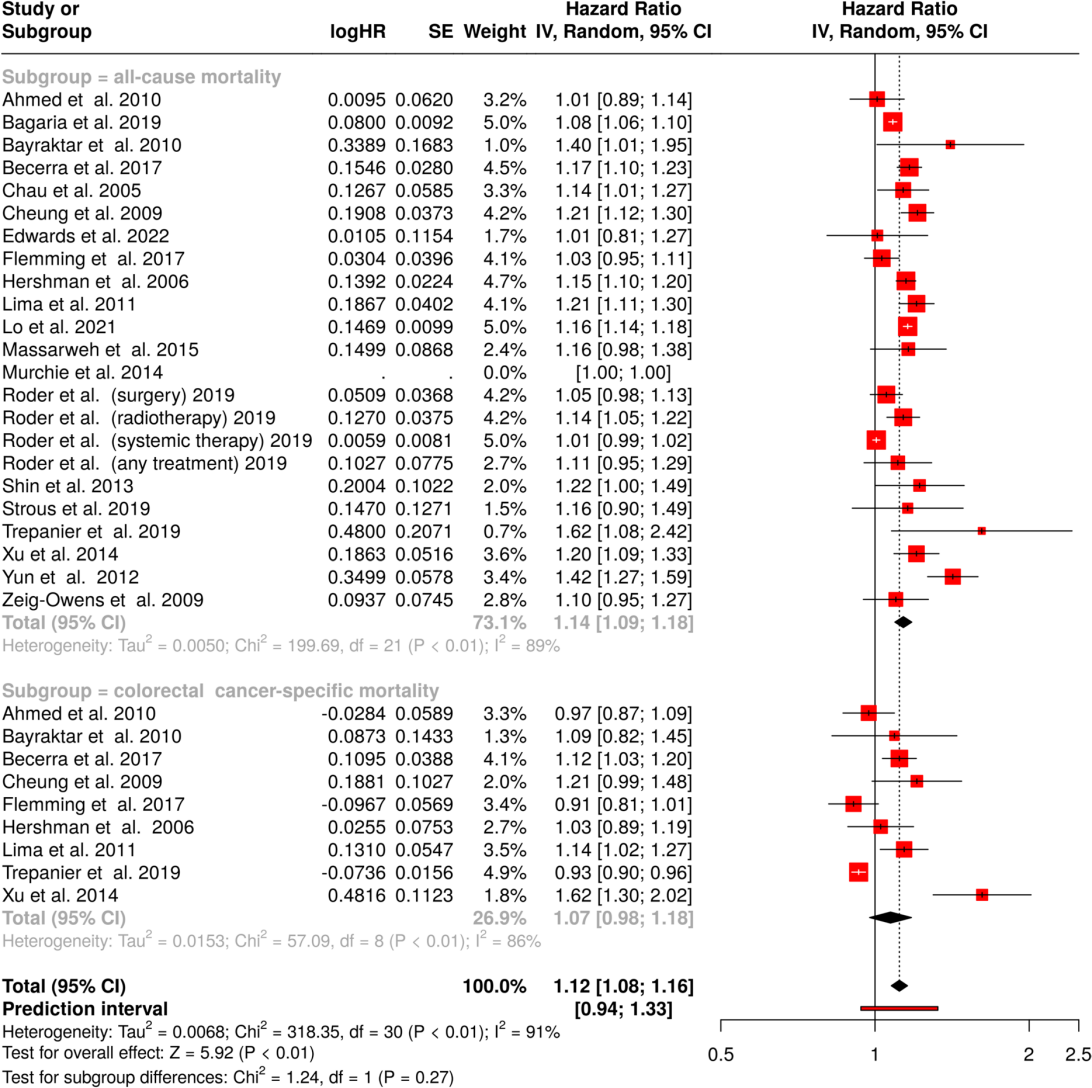
Forest plot depicting the association between a 4-week treatment delay and survival outcomes in colorectal cancer patients. The upper section illustrates the impact on all-cause mortality, while the lower section presents colorectal cancer-specific mortality. Hazard ratios (HR) and corresponding confidence intervals (CI) are displayed for each included study, with pooled estimates calculated using a random-effects model. Squares represent individual study estimates, with their size reflecting study weight, while horizontal lines indicate confidence intervals. The diamond represents the pooled effect estimate. Measures of heterogeneity are reported for both subgroups. CI, confidence interval; HR, hazard ratio; IV, inverse variance; SE, standard error

A 4-week treatment delay was associated with a statistically significant reduction in overall survival, with a pooled HR of 1.14 (95% confidence interval [CI], 1.09–1.18) (Fig. 2, upper panel**)**. Statistical assessment demonstrated significant heterogeneity among studies (*p* < 0.01), with an *I*^*2*^-value of 89%, indicating that most of the heterogeneity between studies was attributable to true heterogeneity rather than random chance variation.

For colorectal cancer-specific mortality, the analysis resulted in a HR of 1.07 (95% CI, 0.98–1.18), indicating no statistically significant impact of delayed treatment on cancer-specific survival (Fig. 2, lower panel). Substantial heterogeneity was evident (*p* < 0.01), with an *I*^*2*^-value of 86%, demonstrating considerable variation in effect sizes across studies. The pooled analysis demonstrated a statistically significant effect with an HR of 1.12 (95% CI, 1.08–1.16). This analysis also revealed significant heterogeneity (*p* < 0.01), with an *I*^*2*^-value of 91%, suggesting substantial true differences in effect sizes across the included studies.

**Fig. 3:**
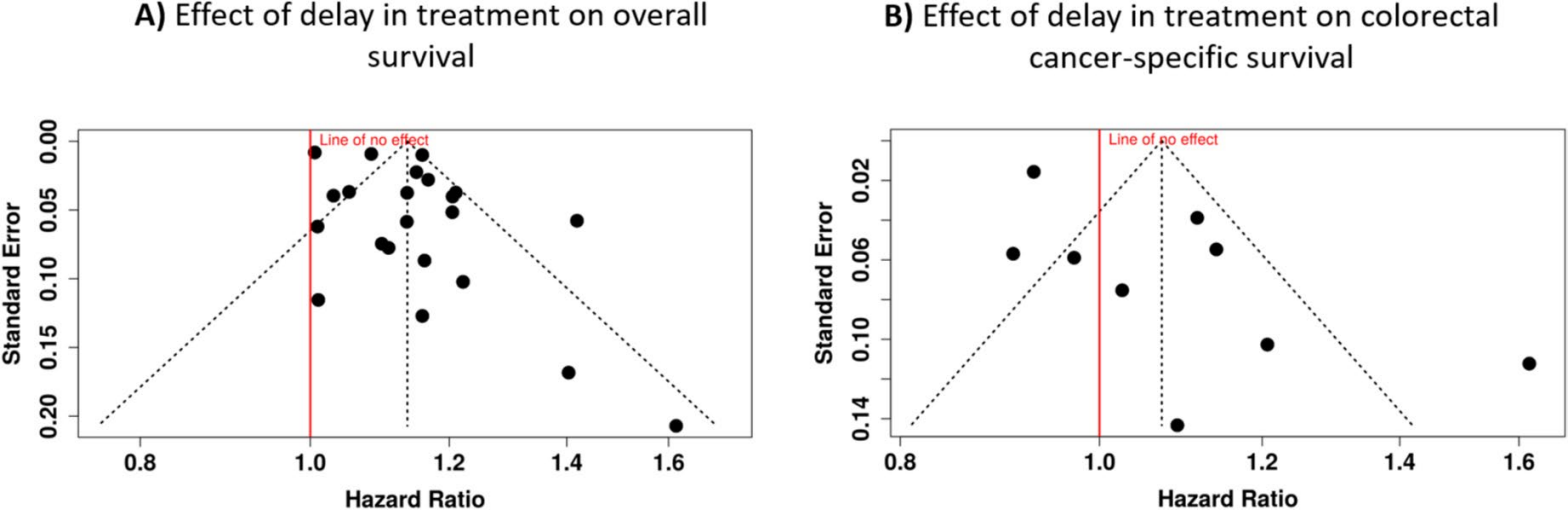
Funnel plots assessing potential publication bias in studies examining the impact of a 4-week treatment delay on colorectal cancer survival. **A** The relationship between overall survival and hazard ratio (HR); **B** colorectal cancer-specific survival. Each dot represents an individual study, with standard error plotted against hazard ratio

Publication bias was thoroughly assessed through multiple methods. The funnel plot analysis for overall survival (Fig. 3A) showed no indication of publication bias, a finding supported by Egger’s test results (intercept, 1.64; 95% CI, − 0.02 and 3.31; *t* = 1.937; *p* = 0.067). However, examination of cancer-specific survival revealed a potential publication bias through funnel plot visualization (Fig. 3B), which was corroborated by Egger’s test findings (intercept, 2.82; 95% CI, 0.72–4.91; *t* = 2.635; *p* = 0.034).

### Effect of 8-week delay in treatment

**Fig. 4:**
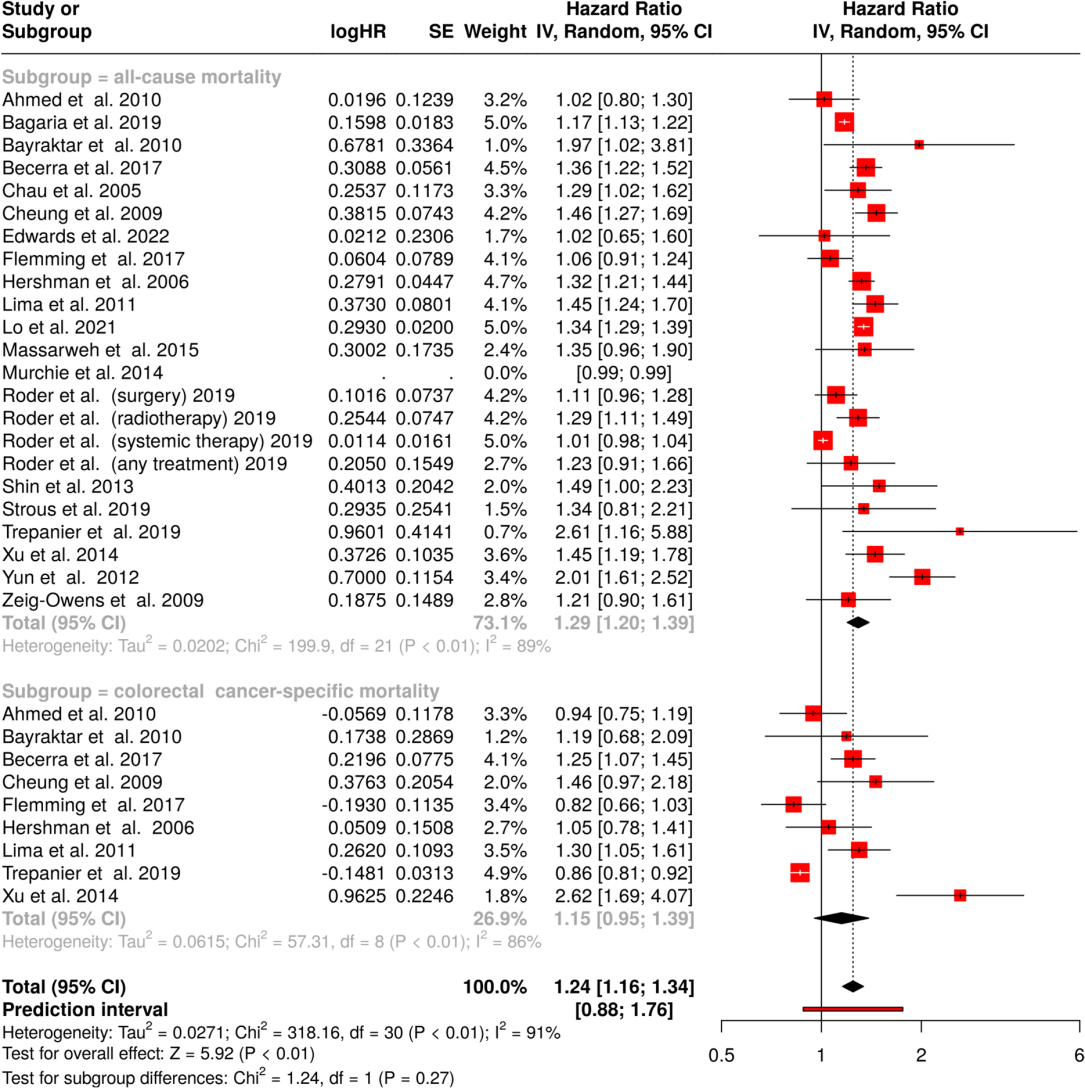
Forest plot evaluating the impact of an 8-week treatment delay on survival outcomes in colorectal cancer patients. The top panel illustrates the association with all-cause mortality, and the lower panel presents the effect on colorectal cancer-specific mortality. CI, confidence interval; HR, hazard ratio; IV, inverse variance; SE, standard error

An increased, statistically significant impact on overall survival was observed for an 8-week treatment delay, with a pooled HR of 1.29 (95% CI, 1.20–1.39), as shown in Fig. 4 (upper panel). Significant heterogeneity was present across studies (*p* < 0.01), with an *I*^*2*^-value of 89%, indicating substantial variation in treatment effect estimates.

For colorectal cancer-specific survival, the analysis resulted in a non-significant effect with an HR of 1.15 (95% CI, 0.95–1.39). Heterogeneity remained significant (*p* < 0.01) with an *I*^*2*^-value of 86%, suggesting considerable variation in effect sizes (Fig. 4, bottom panel). The comprehensive evaluation of all 31 cohorts yielded a statistically significant HR of 1.24 (95% CI, 1.16–1.34), with persistent high heterogeneity (*p* < 0.01; *I*^*2*^ = 91%).

### Effect of 12-week delay in treatment

**Fig. 5:**
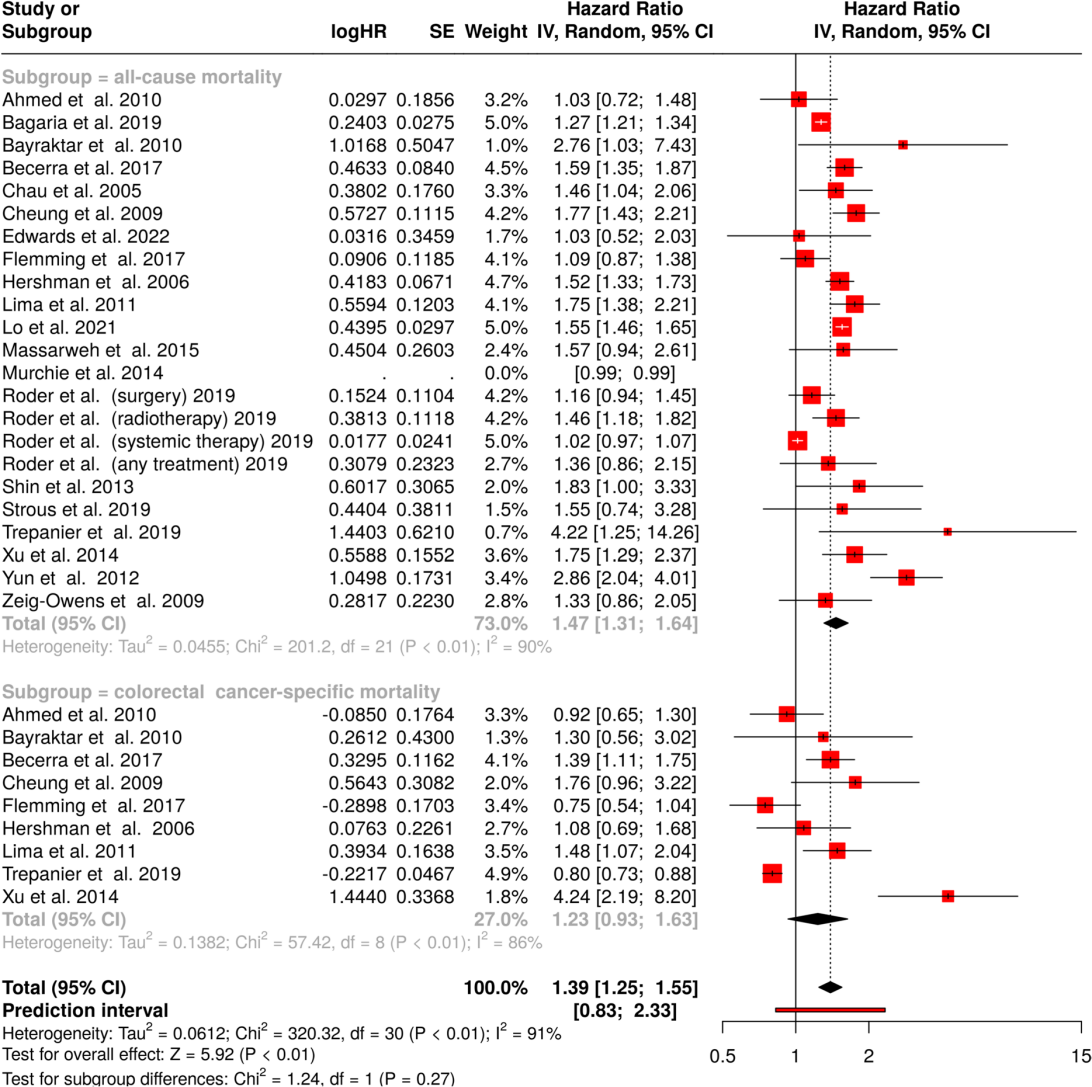
Impact of a 12-week treatment delay on survival in colorectal cancer patients, with all-cause mortality displayed in the upper panel and colorectal cancer-specific mortality in the lower panel. A 12-week treatment delay is associated with a significantly increased risk of mortality, with a pooled hazard ratio of 1.39 (95% confidence interval, 1.25–1.55), indicating a 39% higher risk of death compared to timely treatment, with substantial heterogeneity observed (*I* [2] = 91%). CI, confidence interval; HR, hazard ratio; IV, inverse variance; SE, standard error

The largest increase in mortality risk was observed for a 12-week treatment delay with an HR of 1.47 (95% CI, 1.31–1.64), as depicted in the upper panel of Fig. 5. Statistical assessment showed significant heterogeneity (*p* < 0.01) with an I^2^-value of 90%, indicating considerable variation in effect sizes across studies.

For colorectal cancer-specific survival, the wide confidence interval (95% CI, 0.93–1.63) indicates no statistically significant difference in cancer-specific mortality (Fig. 5, lower panel). Heterogeneity remained significant (*p* < 0.01) with an ^I2^-value of 86%, demonstrating substantial variation in treatment effects. The consolidated analysis of all cohorts revealed a statistically significant effect, with an HR of 1.39 (95% CI, 1.25–1.55). Significant heterogeneity persisted (*p* < 0.01) with an *I*^*2*^-value of 91%, indicating substantial true differences in effect sizes across studies.

## Discussion

This meta-analysis provides compelling evidence that treatment delays in CRC are associated with significantly increased mortality, with progressively worse survival outcomes observed at 4, 8, and 12 weeks of delay. These findings align with prior studies suggesting that delayed initiation of cancer therapy contributes to tumor progression, increased disease burden at treatment onset, and reduced therapeutic efficacy [4, 9].

The impact of treatment delay on CRC outcomes is multifaceted. Delays allow tumors to progress to more advanced stages, increasing the likelihood of lymphovascular invasion [51], perineural invasion [52], and distant metastases. Therefore, minimizing treatment delays is essential to prevent disease progression and improve survival rates in CRC patients. Additionally, delayed treatment can lead to worsened patient performance status, limiting therapeutic options and increasing treatment-related complications.

Delays in CRC treatment arise from a multifactorial interplay of patient-, provider-, and system-related barriers, which often act synergistically to prolong time-to-treatment [10, 53]. Many patients experience delays due to knowledge shortage and low symptom awareness, particularly in early-stage CRC, where signs such as intermittent gastrointestinal discomfort or changes in bowel habits may be misattributed to benign conditions [18, 54, 55]. Psychological factors—including fear of cancer diagnosis, unwillingness to accept colonoscopy [53], concerns about treatment side effects, and reluctance to seek medical attention—further contribute to delays. Socioeconomic challenges such as financial hardship, access to health services, insurance issues, insufficient social support, lack of transportation, visits to alternative medicine practitioners, and inadequate healthcare literacy are also critical factors, disproportionately affecting underserved populations [16, 53, 54].

Delays at the provider level often stem from misdiagnosis or failure to recognize early warning signs, especially in younger patients or those without classic CRC risk factors [56]. In many cases, diagnostic workups are prolonged due to stepwise testing protocols, where patients undergo multiple rounds of noninvasive tests before referral for definitive colonoscopy [56, 57]. Sampling error for biopsy specimens also frequently results in delays [58]. Furthermore, treatment hesitancy—particularly in medically complex patients—may lead to prolonged pre-treatment optimization efforts to ensure they can tolerate aggressive cancer therapies. While these precautions aim to enhance patient safety, they may inadvertently postpone initiation of curative therapy, potentially impacting overall outcomes.

The most substantial delays often arise from structural inefficiencies in healthcare systems [23, 59]. Long wait times for specialist referrals, delays in diagnostic procedures (e.g., colonoscopies, imaging, pathology reports), and bottlenecks in scheduling oncology consultations are persistent issues [20, 60, 61]. These barriers are exacerbated in overburdened healthcare systems with limited resources, particularly in regions with shortages of gastroenterologists, oncologists, and surgical teams. Geographic disparities in healthcare access further contribute to treatment delays, disproportionately affecting patients in rural or underserved areas.

The COVID- 19 pandemic further highlighted the fragility of healthcare systems, with widespread disruptions in elective surgeries, diagnostic procedures, and oncology care, leading to unprecedented delays in cancer treatment worldwide [27, 28, 62, 63]. While some healthcare systems have recovered, residual delays persist, emphasizing the need for resilient, adaptable oncology care pathways that can withstand future crises.

Although CRC affects a broad patient population, older adults are disproportionately impacted by treatment delays due to unique vulnerabilities associated with aging. Age-related frailty [64], multimorbidity [65], and cognitive impairment complicate treatment decision-making, often requiring additional pre-treatment assessments that extend time-to-treatment [66]. Polypharmacy and preexisting conditions may further delay therapy, as oncologists and surgeons navigate potential drug interactions, cardiovascular risk assessments, and anesthesia considerations [67]. Beyond biological factors, older adults also face greater logistical challenges in accessing timely care. Many rely on caregivers for transportation, appointment scheduling, and treatment coordination, which can create additional barriers, particularly for those with limited social support [68]. Moreover, provider biases regarding treatment tolerability in older patients (“age bias”) may contribute to therapeutic nihilism, where aggressive treatments are postponed or withheld due to concerns over toxicity, despite evidence suggesting that appropriately selected older patients benefit from standard therapies [69]. Given the aging global population and the rising burden of CRC in older adults, addressing these vulnerabilities is critical to reducing disparities in cancer treatment outcomes. Strategies such as integrated geriatric-oncology care models, prehabilitation programs, and enhanced multidisciplinary decision-making frameworks can help optimize treatment pathways for older patients while minimizing unnecessary delays [70].

Despite growing evidence that delayed CRC treatment significantly worsens survival, there remains no universally accepted threshold defining an “acceptable” delay [71]. This lack of standardization contributes to substantial variability in clinical practice, with treatment timelines differing across healthcare systems, institutions, and geographic regions. Establishing evidence-based time-to-treatment benchmarks is crucial to ensuring equitable and timely cancer care delivery, particularly for vulnerable patient populations. To address these challenges, policymakers and healthcare institutions must prioritize key reforms aimed at reducing delays and improving patient outcomes. First, streamlining referral pathways by implementing direct-to-specialist triage systems can help expedite diagnostic workups, ensuring that high-risk patients are promptly identified and receive timely intervention [21]. Additionally, expanding access to oncology services, particularly in resource-limited settings, is critical. This can be achieved through telemedicine consultations [72–75], mobile screening programs, and the development of regional cancer networks [76, 77], which would help bridge the gap between urban and rural healthcare infrastructures. Beyond structural improvements, patient-centered strategies must also be implemented to minimize delays. Patient navigation programs—which provide guidance and support to individuals facing logistical, financial, and administrative barriers—have been shown to improve timely access to care, particularly in underserved communities [78, 79]. Finally, integrating comprehensive geriatric assessments into oncology workflows is essential to ensuring that older adults receive timely, personalized, and evidence-based treatment decisions [70]. By systematically evaluating frailty, comorbidities, and functional status, clinicians can make informed treatment choices that balance oncologic urgency with individual patient needs, preventing unnecessary delays while optimizing outcomes. By adopting these multifaceted interventions, healthcare systems can move toward a more standardized, patient-centric approach to CRC treatment, ultimately improving survival rates and reducing disparities in cancer care delivery.

While this meta-analysis provides quantitative evidence that treatment delays significantly increase mortality risk in CRC, further research is needed to refine our understanding of how these delays affect different patient populations and to develop targeted interventions. Future studies should focus on stratifying the risks associated with treatment delays based on key patient characteristics, including age, tumor stage, and comorbidity burden, to create personalized treatment urgency models. Such models could help clinicians prioritize high-risk patients and tailor treatment strategies accordingly. In addition to patient-specific risk assessments, research should examine healthcare system interventions that may mitigate delays. Initiatives such as fast-track diagnostic clinics and multidisciplinary pre-treatment assessments could help streamline care delivery, reduce bottlenecks in oncology services, and ultimately improve patient outcomes [80]. Evaluating the effectiveness of these interventions across different healthcare systems will be crucial for developing evidence-based policies aimed at reducing time-to-treatment disparities. Beyond survival outcomes, future studies should explore the broader consequences of treatment delays, including quality of life, post-treatment functional status, and patient-reported experiences [81, 82]. These factors are particularly relevant for older adults, who may experience greater long-term functional impairment following delayed treatment. Understanding the full spectrum of impacts will help guide patient-centered oncology care and ensure that interventions address both survival and post-treatment well-being. Finally, it is essential to assess real-world data from diverse healthcare settings, particularly in lower-income and rural populations, where barriers to timely treatment are often more pronounced [28]. Expanding research to include these underrepresented groups will provide a more comprehensive, globally applicable understanding of treatment delays and inform policy decisions aimed at reducing disparities in cancer care. By addressing these critical gaps, future research can help refine clinical guidelines, optimize healthcare delivery, and improve outcomes for all patients facing treatment delays in colorectal cancer. While our meta-analysis focused on treatment initiation delays, the impact of chemotherapy interruptions or premature discontinuation—particularly relevant in older adults—also warrants investigation. Emerging data suggest such disruptions may negatively affect survival, and this remains an area for further study.

Although we considered study design, outcome definition clarity, and adjustment for confounders during selection, we did not apply a formal risk of bias assessment tool. We acknowledge this as a limitation and recommend that future meta-analyses incorporate standardized quality assessments, such as the Newcastle––Ottawa Scale. Most studies did not stratify results by cancer stage, limiting our ability to evaluate whether treatment delay has differential effects across early- vs. late-stage CRC. Stage-specific analyses represent an important area for future investigation. While we did not perform formal sensitivity analyses (e.g., excluding outlier studies), informal inspection showed no single study significantly influenced the pooled estimates. We recommend that future work includes predefined sensitivity analyses to further validate findings. Although publication bias was assessed using funnel plots and Egger’s regression, we did not apply Trim-and-Fill or other imputation methods. Incorporating such analyses in future work could enhance detection of small-study effects and potential reporting bias. We did not conduct meta-regression due to limited covariate data across studies. Nevertheless, we recognize that meta-regression could help identify contributors to heterogeneity and should be considered in future reviews. This review was not prospectively registered, which we acknowledge as a limitation. Prospective registration through platforms like PROSPERO would improve transparency and reduce risk of bias and should be pursued in future meta-analyses.

In conclusion, this meta-analysis demonstrates that treatment delays in CRC are associated with significantly worse survival outcomes, reinforcing the importance of timely intervention. Delays arise from a complex interplay of patient, provider, and system-level barriers, with older adults facing additional vulnerabilities that increase their risk of delayed care. Addressing these challenges requires a multidimensional approach, including streamlined healthcare pathways, improved resource allocation, targeted patient support programs, and standardized treatment benchmarks. Future research should further refine age- and risk-stratified treatment guidelines to ensure that all patients, regardless of age or healthcare setting, receive timely and effective CRC treatment.
